# Using deep transfer learning to detect scoliosis and spondylolisthesis from x-ray images

**DOI:** 10.1371/journal.pone.0267851

**Published:** 2022-05-02

**Authors:** Mohammad Fraiwan, Ziad Audat, Luay Fraiwan, Tarek Manasreh

**Affiliations:** 1 Department of Computer Engineering, Jordan University of Science and Technology, Irbid, Jordan; 2 Department of Special Surgery, Jordan University of Science and Technology, Irbid, Jordan; 3 Department of Biomedical Engineering, Jordan University of Science and Technology, Irbid, Jordan; Vellore Institute of Technology: VIT University, INDIA

## Abstract

Recent years have witnessed wider prevalence of vertebral column pathologies due to lifestyle changes, sedentary behaviors, or injuries. Spondylolisthesis and scoliosis are two of the most common ailments with an incidence of 5% and 3% in the United States population, respectively. Both of these abnormalities can affect children at a young age and, if left untreated, can progress into severe pain. Moreover, severe scoliosis can even lead to lung and heart problems. Thus, early diagnosis can make it easier to apply remedies/interventions and prevent further disease progression. Current diagnosis methods are based on visual inspection by physicians of radiographs and/or calculation of certain angles (e.g., Cobb angle). Traditional artificial intelligence-based diagnosis systems utilized these parameters to perform automated classification, which enabled fast and easy diagnosis supporting tools. However, they still require the specialists to perform error-prone tedious measurements. To this end, automated measurement tools were proposed based on processing techniques of X-ray images. In this paper, we utilize advances in deep transfer learning to diagnose spondylolisthesis and scoliosis from X-ray images without the need for any measurements. We collected raw data from real X-ray images of 338 subjects (i.e., 188 scoliosis, 79 spondylolisthesis, and 71 healthy). Deep transfer learning models were developed to perform three-class classification as well as pair-wise binary classifications among the three classes. The highest mean accuracy and maximum accuracy for three-class classification was 96.73% and 98.02%, respectively. Regarding pair-wise binary classification, high accuracy values were achieved for most of the models (i.e., > 98%). These results and other performance metrics reflect a robust ability to diagnose the subjects’ vertebral column disorders from standard X-ray images. The current study provides a supporting tool that can reasonably help the physicians make the correct early diagnosis with less effort and errors, and reduce the need for surgical interventions.

## Introduction

The spinal column is comprised of 33 small bones called vertebrae, which are classified into five distinct areas; cervical, thoracic, lumbar, sacrum, and coccygeal. It is essential for the human body motion and stability. More importantly, the spinal column provides protection for the spinal cord and nerve roots. The spinal cord is part of the central nervous system (CNS) and is responsible for carrying sense and movement information from and to the brain. Hence, the degeneration of the spine results in a wide range of ailments (e.g., restricted motion, pain, numbness, etc.), and reduces the quality of life in general [1].

Several pathologies can affect the vertebral column. In this paper, we examine two types of degenerative pathologies; Scoliosis and Spondylolisthesis. Scoliosis is a curvature of the thoracic or lumbar spine in the coronal plane (i.e., sideways). It is diagnosed by the specialist using X-ray images of the spine and possibly a Magnetic Resonance Imaging (MRI) to rule out tumors [2]. More specifically, the Cobb angle is measured on the image of the vertebrae column, and a value > 10° indicates scoliosis [3]. In addition, other signs can indicate scoliosis (e.g., uneven shoulders, waist, hip, or ribcages). Scoliosis is a common spinal disorder with a prevalence of 0.47-5.2% depending on the country [2]. For example, it is estimated that 6 to 9 million people in the United States suffer from some degree of scoliosis [4]. Spondylolisthesis is a condition caused by an injured vertebral shipping or slipping forward on the vertebrae directly below it [1]. This is typically categorized into different grades depending on the degree of slippage (e.g., low grade vs high grade) [5]. Spondylolisthesis exhibits a prevalence in adult population of 6% [6], and can cause difficulties in standing and walking, numbness, or weakness in one or both legs [5].

The process of diagnosing the spinal column disorders starts with a physical examination. In this step, the doctor investigates the patient’s medical history, participation in sports/physical activity, and involvement in accidents. Moreover, the back and spine need to be carefully examined for signs of abnormal shape, restricted range of motion, or muscle weakness/spasm. In addition, the examination involves performing posture and gait analysis [5]. Once an initial diagnosis is made, the next step would be radiological examinations. X-ray images of the back provides more information about the structure of the spine and the existence of fractures, infections, or other abnormalities. Whereas, computed tomography (CT) images are useful for inspecting the spinal canal. On the other hand, the magnetic resonance imaging (MRI) technique show the spinal cord and nerve, roots and their surroundings [4, 5]. These imaging tests enable the objective determination of biomechanical features (e.g., Cobb angle) and represent a gold standard for the diagnosis of vertebral column ailments [1]. These images are normally taken laterally or from anterior/posterior view of the patient’s back. However, the measurement accuracy of the biomechanical angles is subjective and depends on the experience of the specialist (i.e., radiologist or orthopediatrician). Moreover, high case workload, stress, urgency, or lack of qualified specialists can lead to errors and incorrect diagnosis.

The medical literature in relation to the health of the vertebral column has focused primarily on extracting biomechanical parameters that objectively determine and quantify the disease state of the spine. To this end, scoliosis and its severity can be diagnosed using the Cobb angle, which was described by John Cobb in 1948 and represent the gold standard. However, it has some shortcomings relating to measurement difficulties and in relation to 3D deformities [7]. Similarly, spondylolisthesis can be determined from several parameters that can be measured directly from radiographs. Some of these include; sacral slope, lumbar lordosis, and pelvic incidence. Statistical analysis results in the literature showed significant differences of these parameters across different disease states and normal subjects [1].

The research landscape using machine learning (ML) and artificial intelligence (AI) followed a similar path to that of the medical literature by designing algorithms that can automatically extract the aforementioned biomechanical markers of disease from medical images [8–11], which can be utilized by the specialists for diagnosis. Furthermore, these parameters can be utilized as features for AI-based diagnosis by classifying images into healthy and different disease classes [1, 12, 13]. However, the accuracy of such methods is either low [14–16] or highly dependent on the accuracy of measurement of the biomechanical parameters [1, 12, 17]. In contrast, the work in this paper does not require any explicit measurements of any parameters. It relies on the feature extraction capabilities of deep learning convolutional neural networks to automatically determine the disease class of the input X-ray images. Thus, it eliminates compounded errors and the need for multiple diagnosis steps and complex image processing algorithms.

Recently, deep learning AI architectures has enabled more innovation in disease diagnosis from medical images. For example, Mahajan et al. [18, 19] and Raina et al. [20] employed single shot multiBox detector (SSD) in a combination with deep transfer learning models to detect COVID-19 infections from chest x-ray (CXR) images, and achieved high levels of performance in terms of precision (i.e., 93.01%). In the context of scoliosis, Yang et al. [16] used unclothed back images, after bounding the region of interest (i.e., the subject’s back) using faster recurrent convolutional neural network (Faster-RCNN), as input to the Resnet architecture. They reported an average accuracy of 80% for scoliosis screening but the performance was very low using an external validation dataset (i.e., 55.5%-87%). In a similar study, Kokabu et al. [21] used a combination of 3D depth sensors and a custom-made convolutional neural networks (CNN) to measure the Cobb angle from nude back images. Although their study employed additional hardware, the results show very low specificity (42%-78%). More importantly, the author should have reported the absolute percentage error as the dataset contain a varying range of Cobb angles (0°-64°) and the absolute error does not fully reflect the performance of the model (e.g., an error of 5 of 10 is different from an error of 5 of 50). The approach proposed in this paper does not require extra hardware and achieves superior performance.

The Cobb angle is typically measured using X-ray images. Hence, Tan et al. [22] used a combination of image processing techniques and U-net deep learning architecture to determine the location of vertebrae of interest and subsequently measure the Cobb angle. A wide range of approaches for Cobb angle measurement and scoliosis detection by Karpiel et al. [8]. Classification techniques were also used to distinguish various scoliosis-related classes. Wang et al. [15] designed a deep learning model to differentiate between progressive (P) and non-progressive (NP) classes at first clinic visit. Vergari et al. [23] combined CNN with discriminate analysis to determine the type of scoliosis treatment appearing the X-ray image (i.e., brace, spinal implant, or neither). Although their study did not aim to diagnose scoliosis, the authors claim that their work will facilitate the processing of large databases for such research purposes. Colombo et al. [14] used video raster stereography (RST) as an input to supervised and unsupervised machine learning models, and extracted representative features of scoliosis in comparison to healthy subjects. They reported an accuracy range of 84.9%-87.5%. These traditional approaches still rely on explicit feature extraction and image precessing techniques.

A similar path was taken in the literature for spondylolisthesis identification. Neto et al. [24] used non-deep machine learning techniques (e.g., Support Vector Machine) to differentiate healthy subjects from those suffering from spondylolisthesis/Disk herniation. They used X-ray images as an input and extracted six biomechanical attributes that are markers of the disease states and form the features for classification. They achieved an 85.9% maximum accuracy. This methodology of processing X-ray images to extract disease features and using various classical (i.e., non-deep) machine learning algorithms (e.g., multilayer perceptron) and processing techniques (e.g., clustering) was taken by several related works [1, 12, 25, 26]. However, such explicit extraction of measurements and features may complicate usability and can be error prone [27]. Liao et al. [28] proposed automatic spondylolisthesis measurement using CT images as input. The idea of such approaches is that computerized methods can achieve better accuracy in detecting vertebra edges, features, keypoints, or segmental motion angles [27, 29] in a manner that spondylolisthesis can be accurately determined/graded. This literature suffers from the same aforementioned shortcomings it terms of accuracy, explicit processing, or multiple stages of diagnosis.

The contributions of this paper are as follows:

- Develop a reliable artificial intelligence system for the diagnosis of scoliosis and spondylolisthesis based on radiographic X-ray images of the vertebral column. Such a system can provide support for clinical diagnosis decisions, and reduce errors and overhead.
- We collect X-ray images of subjects suffering from scoliosis and spondylolisthesis, as well as healthy ones, as determined by the specialists in the hospital. This dataset will expand and enrich any comparable publicly available datasets, enable the development of automated machine learning and AI algorithms for the detection of vertebrae ailments, and can be used for training and educating medical students, residents, and specialists.
- Investigate several deep learning convolutional neural network models for the classification of scoliosis, spondylolisthesis, and normal X-ray images using transfer learning.
- We evaluate the performance of the deep learning models for three-class (scoliosis vs spondylolisthesis vs normal) and pair-wise classification problems (scoliosis vs spondylolisthesis, scoliosis vs normal, and spondylolisthesis vs normal). The cost of each model in terms of training and testing times were also evaluated.
- We share, through public data repository, the original images and resized versions that match the requirements of deep learning models in five sizes; [224 224 3], [227 227 3], [256 256 3], [299 299 3], and [331 331 3].

The rest of this paper is organized as follows. In the materials and methods section, we present the data collection procedure, subjects, deep learning models, performance evaluation setup, and performance metrics. The results section provides the results in detail and discussion of the various observations. The conclusion section presents the future works and concludes the work in this paper.

## Materials and methods

**Fig 1:**
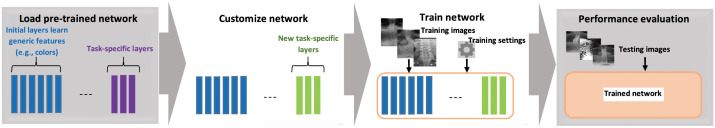
A graphical abstract of the transfer deep learning approach.

The work in this paper exploits the abilities of generically pre-trained convolutional neural network models to automatically classify X-ray images into three possible spine-related conditions; scoliosis, spondylolisthesis, or normal(i.e., healthy). The approach achieves high performance metrics while not requiring manual or automatic measurements nor any feature extraction as this is inherently done by the deep learning architecture. In addition, no elaborate image processing or modeling are required. Fig 1 shows the general steps for customizing the pre-trained models for classification of the X-ray images into normal (i.e., healthy), scoliosis, or spondylolisthesis.

### Subjects and data collection

The current study was conducted according to the guidelines of the Declaration of Helsinki and approved by the Institutional Review Board (IRB) at King Abdullah University Hospital (KAUH), Deanship of Scientific Research at Jordan University of Science and Technology in Jordan (Ref. 19/144/2021). X-ray images of the vertebral column were collected locally at King Abdullah University Hospital, Jordan University of Science and Technology, Irbid, Jordan. Written informed consent was obtained from all subjects involved in the study (or their parents in case of minors). The diagnosis was determined by two orthopedic specialists at the KAUH.

The dataset included 338 subjects (240 females, 98 males) with an age range from 9 months to 79 years and mean ± SD of 24.9 ± 18.58 years. The number of subjects with normal X-ray images was 71 (40 females, 31 males) with an age range of 9 months to 56 years and mean ± SD of 19.41 ± 11.19. The number of subjects diagnosed with spondylolisthesis was 79 (49 females, 30 males) with an age range of 15-79 years and mean ± SD of 53.59 ± 14.02. The number of subjects diagnosed with scoliosis was 188 (151 females, 37 males) with an age range of 5-35 years and mean ± SD of 14.73 ± 3.36.

### Deep learning models

Typically, the main input to the diagnosis of vertebral column diseases is medical images (i.e., X-ray, CT, or MRI). Hence, convolutional neural networks (CNNs) were used to classify the input into the possible disease state. CNNs are a type of feed forward neural networks with a deep architecture and form the basis for a major part of the deep learning models (DNNs) in the literature. Other types include Recurrent Neural Network (RNN) with variations (e.g., Long Short Term Memory (LSTM), and transformers), and Generative adversarial networks (GANs). CNNs have been found to be useful for image processing and classification as they are able to extract patterns and features in images regardless of scaling, mirroring, rotation, or translation.

The CNN is generally comprised of several types of layers and takes a tensor of order 3 as input (i.e., an image with N rows, M columns, and 3 (RGG) color channels). Convolution layers scan the image looking for correlated regions (e.g., vertebra). The input image is divided into small subparts called receptive fields, which in turn are grouped into feature maps. Each feature map has a corresponding weight matrix (i.e., kernel), which is learned/updated during training. Rectified linear unit (ReLU) usually follows the convolution layer and introduces nonlinearity into the CNN. Pooling layers reduce the dimensionality of the feature maps feeding into subsequent layers by considering subparts of the feature map and taking the maximum (i.e., max-pooling), average (i.e., average-pooling), or other statistical measure. Fully connected layers are similar to multilayer perceptron (MLP) networks and ensure that all elements in the previous layer contribute to the output or following layer. Dropout layers remove certain elements of the network in order to prevent overfitting and improve model generalization. The mathematical foundations, benefits, alternatives, and tradeoffs are well-established in the literature and beyond the scope of this work [30].

Transfer learning utilizes pre-trained deep learning models, which were developed using millions of images from the ImageNet [31] and other databases (e.g., Places365 [32]). The models are able to classify images into hundreds of categories. However, they can be tailored and retrained to preform new tasks using transfer learning. For this to work, the final layer need to be changed to match the number of output classes in the new task. Depending on the model, the final layer could be a FullyConnectedLayer or a Convolution2DLayer, and needs to be replaced accordingly with a number of filters equal to the number of output classes. As for the input, each model requires images to be of a certain dimension (e.g., [244 244 3]), which requires resizing. In addition, grayscale images (i.e., 2D) need to be transformed to rgb (i.e., 3D) images.

The following is a short description of the 14 convolutional neural network models used in this paper:

- SqueezeNet is 18 layers deep with an image input size of [227 227 3]. It was designed with the premise that smaller deep neural networks can offer comparable accuracy levels to large architectures but with the advantages of lesser inter-process communication, faster deployment on end-user machines, and more suitability to resource-limited environments. The model was pre-trained using the ImageNet database [31] to classify images into 1000 possible object classes (e.g., screwdriver, car, etc.). In this paper, SqueezeNet v1.1 was used, which provides the same accuracy as SqueezeNet v1.0 but with less computational overhead [33].
- GoogLeNet is 22 layers deep with an image input size of [224 224 3]. It is part of the family of Inception deep learning models and it is marked by the improved utilization of the computing resources, which allowed for increasing the depth and width of the network without any additional computational cost [34]. The model is available pre-trained on images from ImageNet or Places365 [32]. The former was used in this work.
- Inception-v3 is the third version of the Inception models, which improves on the previous two by having more parameters (e.g., utilizing three different filter sizes in the parallel convolution layers). The model is 48 layers deep with an image input size of [299 299 3] pre-trained on images form ImageNet [35].
- DenseNet-201, as the name suggests, is 201 layers deep with an image input size of [224 224 3]. The model represents a big jump in the number of layers compared to others. This was made possible by shortening the connections between layers close to the input/output. Connections between layers are made such that each layer feeds into later layers, which improves feature propagation/reuse and drastically reduces the number of parameters [36].
- MobileNets is 53 layers deep with an image input size of [224 224 3]. It is a network designed for mobile environments. Thus, the model is required to be efficient and small by reducing the memory requirements. This is achieved by inverted residual bottleneck layers that require computation that can be scheduled with minimum working set (i.e., number of tensors concurrently stored in memory) [37].
- ResNet-101, ResNet-50, and ResNet-18. The ResNet family of models with the corresponding layer depth require the same image input size of [224 224 3] and pre-trained on the ImageNet database. The architecture is characterized by using network-in-network scheme that employ learning residual functions with reference to layer inputs [38]. It is a winner of the ImageNet Large Scale Visual Recognition Challenge 2015 (ILSVRC2015).
- The Xception model is 71 layers deep with an image input size of [299 299 3]. It is trained on images from the ImageNet database. The architecture improves on the Inception network by replacing the standard inception modules with depthwise separable convolutions [39].
- The Inception-ResNet-v2 model is 164 layers deep with an image input size of [299 299 3]. It is trained on images from the ImageNet database. The architecture is hybrid of the Inception model and residual connections, which results in faster training [40].
- ShuffleNet is another model designed for resource limited deployment environments. It is based on pointwise group convolutions and channel shuffling, to drastically improve the computational overhead without scarifying the classification accuracy [41]. The model is pre-trained using the ImageNet database and requires an image input size of [224 224 3].
- NAsnetMobile is the mobile version of the Neural Architecture Search Network (Nasnet) model. The main idea of this type of models is to learn the network architecture during training on the specific dataset using reinforcement learning search. Converging to the best model is reduced to finding the optimal cell structure (i.e., convolutional layer), which is duplicated to other convolutional networks but with different weights [42]. The model is pre-trained on the ImageNet database and requires an image input size of [224 224 3].
- DarkNet-53 is pre-trained on the ImageNet database and requires an input image of size [256 256 3]. The model is 53 layers deep and was designed with speed and object detection as primary objectives [43]. It improves on the previous version, DarkNet-19 by using more layers and employing residual connections [44].
- EfficientNet-b0 is the baseline EfficientNet architecture, which provides scaled models up to EfficientNet-b7. The architecture design is based on the idea of compound scaling, which uniformly scale the network depth, width, and input resolution by fixed scaling coefficients [45]. The model is pre-trained using the ImageNet database and requires an input image of size [224 224 3].

### Performance evaluation setup

The deep learning models were modified, trained, and evaluated using MATLAB R2021a software running on an HP OMEN 30L desktop GT13 with 64 GB RAM, NVIDIA^®^GeForce RTX^™^ 3080 GPU, Intel^®^Core^™^ i7-10700K CPU @ 3.80GHz, and 1TB SSD.

To prevent the models from overfitting specific image details, pixel translation (i.e., shifting the image) by 30 pixels vertically and horizontally was performed on the X-ray images used for training. Moreover, training images were randomly flipped along the x-axis (i.e., reflection), and rescaled from the range [0.9,1.1]. The model training options were set such that the minimum batch size was 10 (except for NASNet-Mobile, which had the size set to 2 due to slowness), the max epochs was set to 6, and initial learning rate was 0.003. Moreover, the stochastic gradient descent with momentum (SGDM) optimizer was used for training due to popularity and fast convergence [46]. The holdout method with a split of 70% training and 30% testing was used. Furthermore, to counter any bias in the data split, the experiments were repeated 40 times, and the minimum, maximum, average, and standard deviation (SD) were reported. In addition, samples of the training and validation curves were reported for the highest preforming model for each classification problem.

### Performance metrics

The performance of the models was evaluated using five metrics: precision, recall, specificity, F1 score, and accuracy. Precision is the ratio of true positives to all images identified as positive (i.e., including false positives). Recall (i.e., sensitivity) is the ratio of true positives to all relevant elements (i.e., the actual positives). Specificity, or the true negative rate, measures the ability to identify negative elements. The F1 score is the harmonic mean of the recall and precision and expresses the accuracy of classification in unbalanced datasets. The accuracy is defined as the ratio of the true positives for all classes to the number of instances (i.e., total images in the testing set). The five measures are defined as follows:

$$\begin{array}{c} Precision=\frac{TP}{TP+FP} \end{array}$$ (1)

$$\begin{array}{c} Recall=\frac{TP}{TP+FN} \end{array}$$ (2)

$$\begin{array}{c} Specificity=\frac{TN}{TN+FP} \end{array}$$ (3)

$$\begin{array}{c} F1=2\times \frac{Recall\times Precision}{Recall+Precision} \end{array}$$ (4)

$$\begin{array}{c} Accuracy=\frac{\sum_{i}^{No.\;of\;Classes}TP_{i}}{No.\;of\;testing\;images} \end{array}$$ (5)

Where *TP* (true positives) is the number of correctly classified images (i.e., for each one of the classes), *FP* (false positives) is the number of wrongly classified images as another class, and *FN* (false negatives) is the number of images missed by the classifier.

## Results and discussion

The purpose of the experiments was to evaluate the effectiveness of the pre-trained models, after customization and training, in identifying the correct disease diagnosis of the X-ray image. Moreover, since deep learning algorithms incur high overhead, the time of the training and testing was recorded too. Depending on the classification problem (three classes or two, and type of disease), the number of testing images ranged from 45 to 101.

Tables 1 and 2 show the performance evaluation metrics for classifying X-ray images into normal, scoliosis, or spondylolisthesis. The DensNet-201 achieved the highest accuracy value over the three statistical measures with a mean of 96.34%, maximum 99.01%, and minimum 94.06%. On the other hand, the baseline EfficientNet model performed the worst with an average accuracy of 87.92%, although NASNet-Mobile scored the lowest minimum accuracy of 78.22%. The later displayed the highest variation in accuracy values based on the standard deviation of 4.8%. The other performance metrics display a consistent and homogenous ability to identify negative as well as positive cases with a similar performance pattern to the accuracy results (i.e., DenseNet-201 achieving the best results). The F1-score is of special importance as the dataset is imbalanced due to the scoliosis class having more images in comparison to the other two. Thus, the accuracy values maybe misleading, but this is not the case as the F1-score reflects a similar performance over all classes.

**Table 1:** The accuracy of classifying X-ray images into three classes; normal, scoliosis, or spondylolisthesis, for each deep learning model. The results are reported for 40 runs of each model. SD stands for standard deviation.

Model	Mean accuracy	Max. accuracy	Min. accuracy	SD
SqueezeNet	91.29%	95.05%	87.13%	2.94%
GoogLeNet	93.76%	96.04%	91.09%	1.40%
Inception-v3	92.97%	95.05%	89.11%	1.83%
DenseNet-201	96.34%	99.01%	94.06%	1.48%
MobileNet-v2	91.39%	95.05%	88.12%	1.75%
ResNet-101	93.27%	95.05%	86.14%	2.71%
ResNet-50	94.36%	96.04%	91.09%	1.98%
ResNet-18	94.26%	95.05%	92.08%	1.02%
Xception	88.22%	92.08%	85.15%	2.58%
Inception-ResNet-v2	90.30%	94.06%	83.17%	3.05%
ShuffleNet	92.38%	96.04%	89.11%	2.38%
NASNet-Mobile	90.30%	95.05%	78.22%	4.80%
DarkNet-53	91.58%	95.05%	86.14%	2.85%
EfficientNet-b0	87.92%	91.09%	83.17%	2.18%

**Table 2:** The mean overall F1 score, precision, and recall parameters for the 14 deep learning models performing three-class classification.

Model	F1 Score	Precision	Recall	Specificity
SqueezeNet	89.98%	94.10%	88.00%	94.54%
GoogLeNet	93.24%	95.52%	91.55%	95.88%
Inception-v3	92.32%	94.66%	90.93%	94.97%
DenseNet-201	95.97%	97.61%	94.62%	97.89%
MobileNet-v2	90.35%	93.74%	88.13%	94.45%
ResNet-101	92.55%	96.15%	90.16%	96.38%
ResNet-50	93.84%	96.74%	91.79%	96.91%
ResNet-18	93.82%	96.65%	91.73%	96.80%
Xception	86.71%	93.08%	83.47%	93.32%
Inception-ResNet-v2	89.34%	92.99%	87.36%	93.29%
ShuffleNet	91.76%	94.48%	90.28%	94.55%
NASNet-Mobile	89.77%	90.99%	89.62%	91.00%
DarkNet-53	90.62%	94.77%	88.23%	95.01%
EfficientNet-b0	86.41%	92.98%	82.62%	93.47%

**Fig 2:**
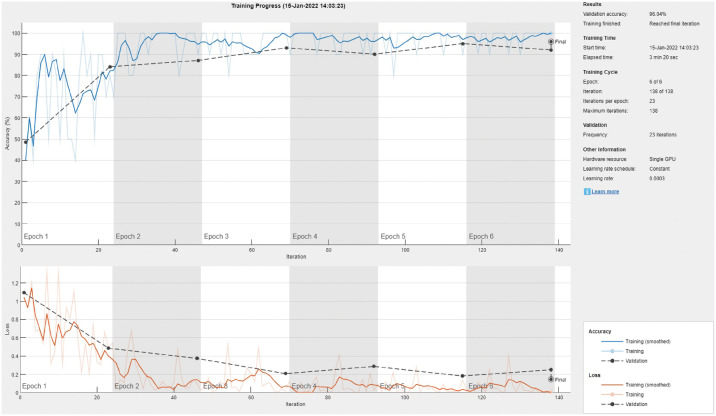
The DenseNet-201 sample training and validation curve for three-class classification.

Fig 2 shows the training and validation progress curve for a sample run of the highest performing model, which gives an indication of the fitting performance of the model and the need for more training/data. The loss value indicates the error while training/validation. The figures show that there is no underfitting as the two validation and training loss curves are going down consistently and within a small gap to each other. Similarly, there is no overfitting as they are not diverging toward the end of the training epochs. Furthermore, the validation curve does not great noisy progress, which means that the validation dataset is representative of the classification problem (i.e., the ability of the model to generalize).

**Fig 3:**
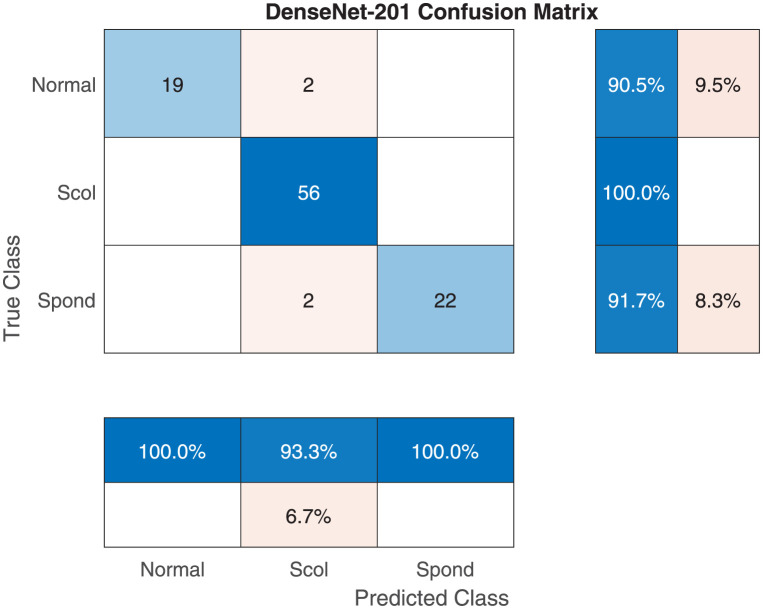
The DenseNet-201 sample confusion matrix for three-class classification.

**Fig 4:**
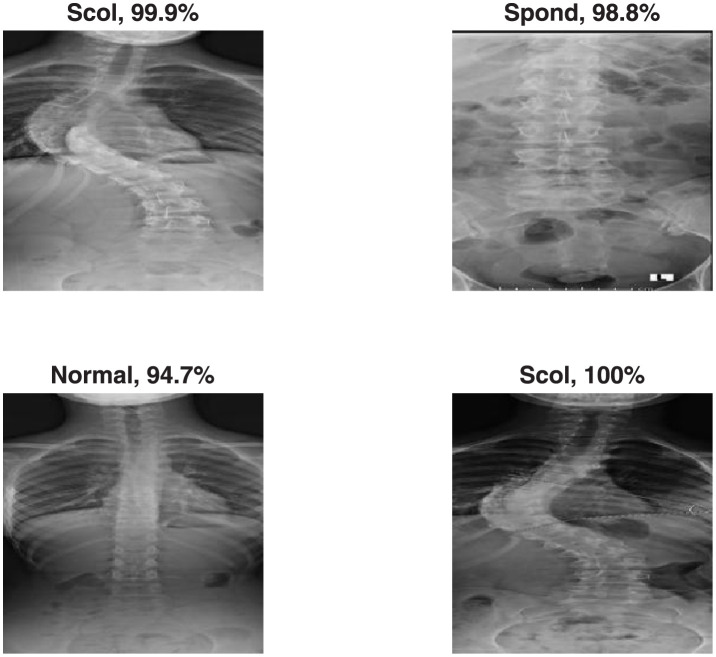
A sample output from the three class classification process.

Fig 3 shows the DenseNet-201 sample confusion matrix for three-class classification. The model performs almost consistently over all classes with scoliosis detected perfectly but 2 normal images misclassified as scoliosis and 2 spondylolisthesis cases misclassified as scoliosis. The number of testing images is 101. Fig 4 shows a sample output from the three class classification process with the identification probability calculated by the deep learning model for each.

**Fig 5:**
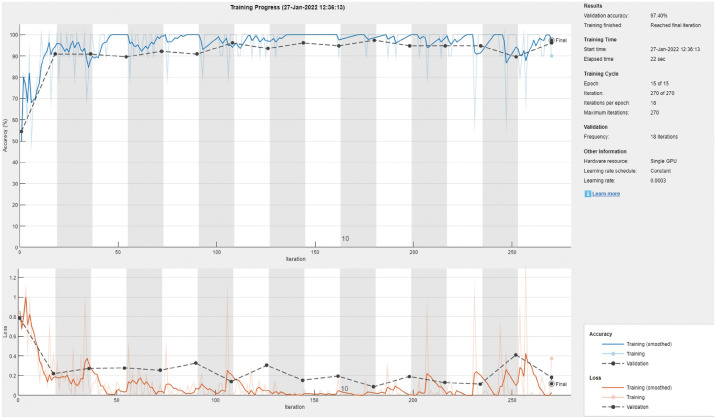
The RestNet-18 sample training and validation curve for normal vs scoliosis classification.

**Fig 6:**
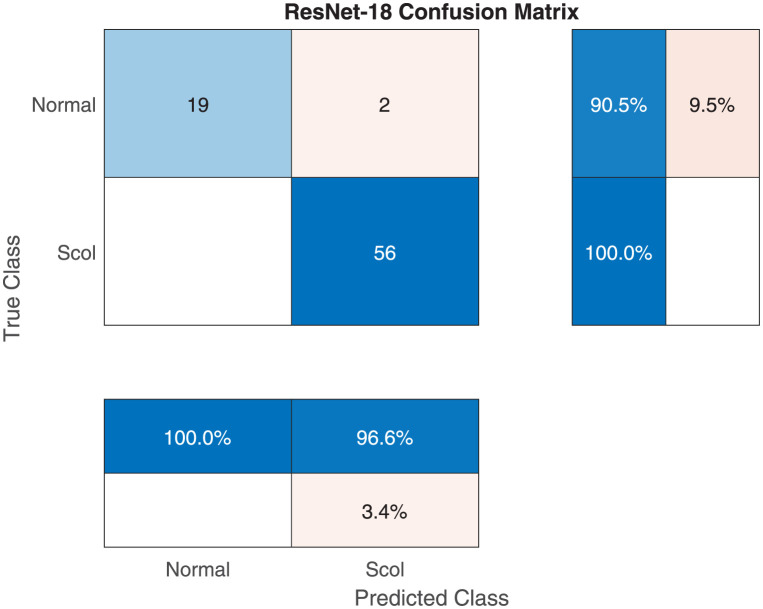
The RestNet-18 sample confusion matrix for normal vs scoliosis classification.

Tables 3 and 4 show the performance evaluation metrics for classifying X-ray images into normal or scoliosis. The Resnet-101 and ResNet-18 achieved the highest mean accuracy (i.e., 97.66%) although the ResNet-18 model is smaller and faster. Since this is an easier classification problem that the three-class one, all models achieved high accuracy values with less standard deviation over multiple runs. However, the NASNet-Mobile model had a 4.55% SD. Similarly, the F1 score and other metrics display consistent good performance over all classes. Fig 5 shows a sample training and validation progress curve showing the loss and accuracy values. The figure clearly displays a stable learning behavior and appropriate training and validation sets. Fig 6 shows the confusion matrix for a sample run of ResNet-18. In that run, there were no false negatives but 2 false positive cases. The number of testing images is 77.

**Table 3:** The accuracy of classifying X-ray images into two classes; normal or scoliosis, for each deep learning model. The results are reported for 40 runs of each model.

Model	Mean accuracy	Max. accuracy	Min. accuracy	SD
SqueezeNet	95.71%	98.70%	92.21%	2.13%
GoogLeNet	97.01%	97.40%	93.51%	1.23%
Inception-v3	96.23%	97.40%	93.51%	1.43%
DenseNet-201	97.01%	98.70%	96.10%	0.88%
MobileNet-v2	95.45%	97.40%	94.81%	0.92%
ResNet-101	97.66%	98.70%	97.40%	0.55%
ResNet-50	97.14%	97.40%	96.10%	0.55%
ResNet-18	97.66%	98.70%	96.10%	1.02%
Xception	91.17%	94.81%	88.31%	2.36%
Inception-ResNet-v2	93.38%	96.10%	88.31%	2.16%
ShuffleNet	95.97%	98.70%	90.91%	2.25%
NASNet-Mobile	92.73%	97.40%	83.12%	4.55%
DarkNet-53	97.27%	98.70%	96.10%	0.74%
EfficientNet-b0	92.99%	96.10%	88.31%	2.46%

**Table 4:** The mean overall F1 score, precision, and recall parameters for the 14 deep learning models performing normal vs scoliosis classification.

Model	F1 Score	Precision	Recall	Specificity
SqueezeNet	94.33%	96.26%	93.04%	97.50%
GoogLeNet	96.18%	97.52%	95.12%	98.32%
Inception-v3	95.01%	97.38%	93.24%	98.28%
DenseNet-201	96.12%	97.69%	94.82%	98.48%
MobileNet-v2	93.97%	96.69%	91.96%	97.84%
ResNet-101	96.97%	98.45%	95.71%	98.98%
ResNet-50	96.27%	98.11%	94.76%	98.75%
ResNet-18	97.01%	97.80%	96.31%	98.55%
Xception	87.54%	93.54%	84.55%	95.86%
Inception-ResNet-v2	90.90%	95.24%	88.30%	96.92%
ShuffleNet	94.70%	96.37%	93.66%	97.56%
NASNet-Mobile	89.45%	95.41%	86.82%	96.98%
DarkNet-53	96.51%	97.29%	95.89%	98.20%
EfficientNet-b0	90.34%	94.76%	87.74%	96.63%

**Fig 7:**
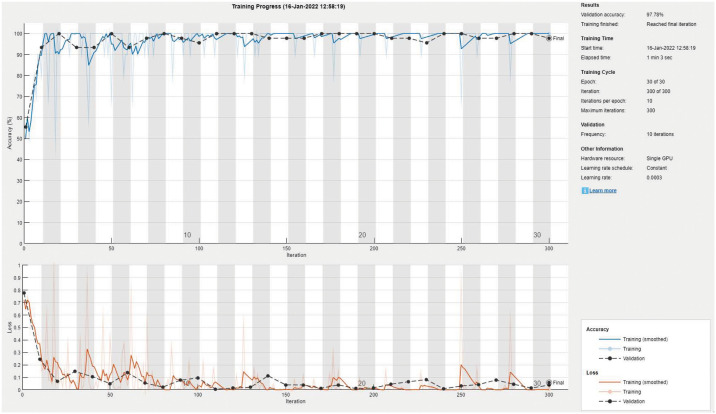
The RestNet-101 sample training and validation curve for normal vs spondylolisthesis classification.

**Fig 8:**
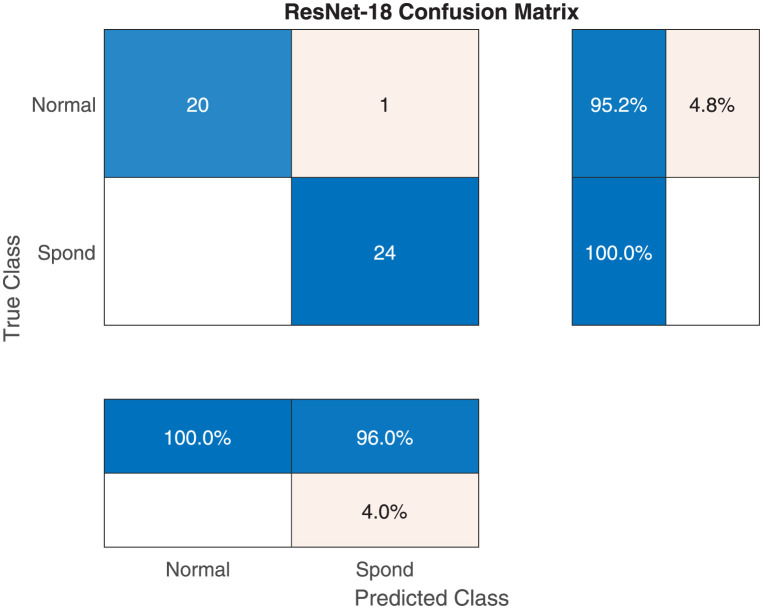
The RestNet-101 sample confusion matrix for normal vs spondylolisthesis classification.

Tables 5 and 6 show the performance evaluation metrics for classifying X-ray images into normal or spondylolisthesis. Most models achieved very high mean accuracy (> 96%) with ResNet-101 achieving the highest value of 99.33%. Several models achieved a maximum accuracy of 100%, however the NASNet-Mobile model achieved the lowest accuracy with high fluctuation over several runs (5.18% SD) along with the DarkNet-53 model (4.94% SD). Fig 7 shows an excellent training/validation progress curve with the training and validation losses decrease to a point of stability with a very small gap between them (i.e., no overfitting/underfitting). Fig 8 shows a sample confusion matrix with one false positive case (i.e., normal diagnosed as spondylolisthesis). The number of testing images is 45.

**Table 5:** The accuracy of classifying X-ray images into two classes; normal or spondylolisthesis, for each deep learning model. The results are reported for 40 runs of each model.

Model	Mean accuracy	Max. accuracy	Min. accuracy	SD
SqueezeNet	98.00%	100.00%	95.56%	1.95%
GoogLeNet	96.00%	100.00%	93.33%	2.93%
Inception-v3	96.22%	100.00%	91.11%	2.78%
DenseNet-201	98.00%	100.00%	95.56%	1.64%
MobileNet-v2	96.22%	97.78%	93.33%	1.83%
ResNet-101	99.33%	100.00%	97.78%	1.07%
ResNet-50	98.44%	100.00%	97.78%	1.07%
ResNet-18	98.22%	100.00%	97.78%	0.94%
Xception	96.00%	97.78%	91.11%	2.73%
Inception-ResNet-v2	96.22%	100.00%	95.56%	1.50%
ShuffleNet	97.56%	100.00%	95.56%	1.64%
NASNet-Mobile	86.44%	93.33%	80.00%	5.18%
DarkNet-53	96.89%	100.00%	84.44%	4.94%
EfficientNet-b0	96.44%	97.78%	88.89%	2.81%

**Table 6:** The mean overall F1 score, precision, and recall parameters for the 14 deep learning models performing normal vs spondylolisthesis classification.

Model	F1 Score	Precision	Recall	Specificity
SqueezeNet	98.00%	98.01%	98.12%	97.87%
GoogLeNet	95.99%	96.27%	96.16%	95.82%
Inception-v3	96.21%	96.31%	96.25%	96.18%
DenseNet-201	97.98%	98.17%	97.89%	98.11%
MobileNet-v2	96.18%	96.62%	96.04%	96.41%
ResNet-101	99.33%	99.37%	99.32%	99.34%
ResNet-50	98.43%	98.60%	98.33%	98.55%
ResNet-18	98.21%	98.40%	98.10%	98.34%
Xception	95.96%	96.36%	95.89%	96.11%
Inception-ResNet-v2	96.19%	96.55%	96.07%	96.37%
ShuffleNet	97.54%	97.73%	97.47%	97.65%
NASNet-Mobile	85.84%	89.39%	85.68%	87.38%
DarkNet-53	96.84%	97.13%	96.79%	97.00%
EfficientNet-b0	96.43%	96.64%	96.37%	96.51%

**Fig 9:**
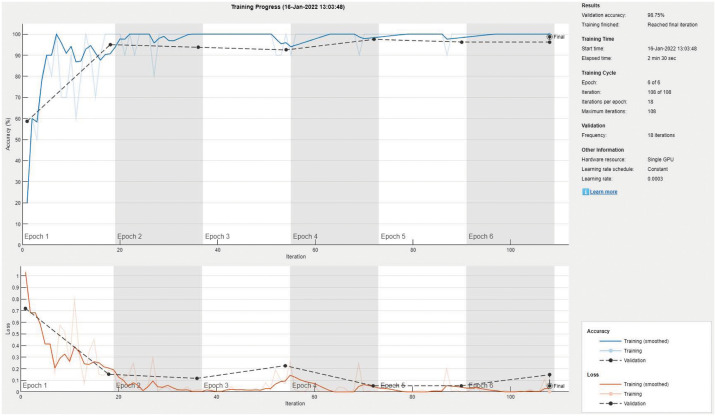
The DenseNet-201 sample training and validation curve for scoliosis vs spondylolisthesis classification.

**Fig 10:**
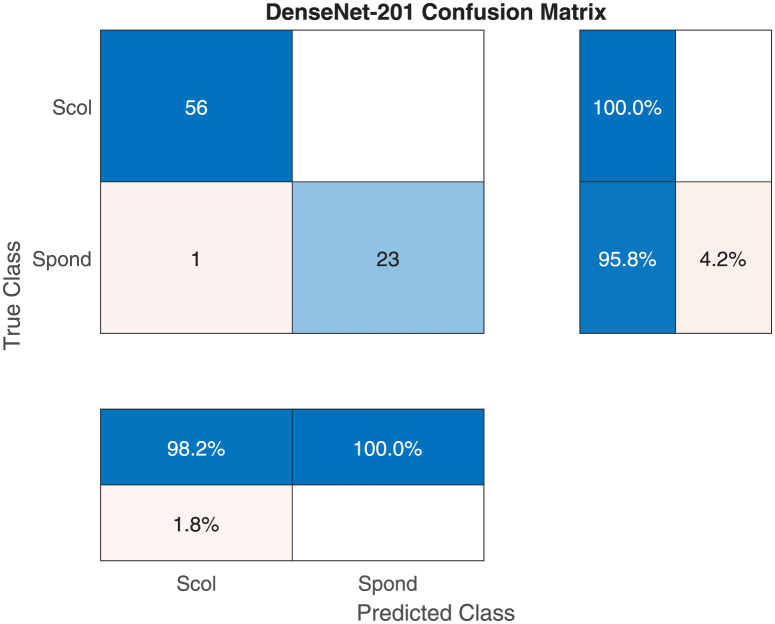
The RestNet-101 sample confusion matrix for scoliosis vs spondylolisthesis classification.

Tables 7 and 8 show the performance valuation metrics for classifying X-ray images into scoliosis vs spondylolisthesis. The performance of all models drops, although with varying degrees, as they try to differentiate between two disease states. Nonetheless, Dense-Net-101 achieved a high mean accuracy of 97%. One notable difference from the other classification results is that some models achieved a low minimum accuracy (Inception-ResNet-v2: 78.75% and 4.97% SD, NASNet-Mobile: 73.75% and 6.12% SD). In addition, almost all models displayed greater standard deviation. This indicates the sensitivity of the results to the type of training/validation data split in most models. Fig 9 shows the training/validation curve for the DenseNet-201 model. The figure displays a stable learning curve. Fig 10 shows a sample confusion matrix with one spondylolisthesis case misdiagnosed as scoliosis. The number of testing images is 80.

**Table 7:** The accuracy of classifying X-ray images into two classes; scoliosis, or spondylolisthesis, for each deep learning model. The results are reported for 40 runs of each model.

Model	Mean accuracy	Max. accuracy	Min. accuracy	SD
SqueezeNet	93.88%	97.50%	86.25%	3.14%
GoogLeNet	94.75%	96.25%	91.25%	1.75%
Inception-v3	94.00%	98.75%	88.75%	3.43%
DenseNet-201	97.00%	98.75%	96.25%	1.05%
MobileNet-v2	92.62%	97.50%	88.75%	2.73%
ResNet-101	93.12%	95.00%	90.00%	1.89%
ResNet-50	94.50%	96.25%	90.00%	2.30%
ResNet-18	95.13%	97.50%	92.50%	1.90%
Xception	88.38%	92.50%	86.25%	2.13%
Inception-ResNet-v2	89.50%	95.00%	78.75%	4.97%
ShuffleNet	93.62%	96.25%	90.00%	2.24%
NASNet-Mobile	89.00%	96.25%	73.75%	6.12%
DarkNet-53	93.75%	98.75%	88.75%	2.50%
EfficientNet-b0	91.25%	93.75%	90.00%	1.32%

**Table 8:** The mean overall F1 score, precision, and recall parameters for the 14 deep learning models performing scoliosis vs spondylolisthesis classification.

Model	F1 Score	Precision	Recall	Specificity
SqueezeNet	92.06%	95.91%	89.91%	97.00%
GoogLeNet	93.39%	96.23%	91.49%	97.25%
Inception-v3	92.22%	96.00%	90.12%	97.05%
DenseNet-201	96.31%	97.95%	95.00%	98.5%
MobileNet-v2	90.66%	93.66%	88.90%	95.44%
ResNet-101	91.15%	95.40%	88.66%	96.63%
ResNet-50	92.98%	96.40%	90.83%	97.35%
ResNet-18	93.88%	96.45%	92.11%	97.42%
Xception	84.05%	92.91%	80.62%	94.86%
Inception-ResNet-v2	85.44%	93.03%	82.98%	94.87%
ShuffleNet	92.09%	94.19%	90.92%	95.69%
NASNet-Mobile	87.72%	87.86%	89.76%	88.28%
DarkNet-53	91.95%	95.95%	89.58%	97.02%
EfficientNet-b0	88.61%	93.88%	85.77%	95.58%

Since deep learning models are computation intensive, we have compared the time required to train and test each model. Table 9 shows the mean training and validation times for each of the 14 deep learning models for the four types of classification problems in this work. As the table shows, the smaller the dataset, the lesser the time required by all models. SqueezeNet required the least time and it is very fast in comparison to all others. However, the time required by the highest accuracy models (DenseNet-201, ResNet-18, and ResNet-101) is somewhat reasonable. On the other hand, NasNet-Mobile is extremely slow and achieved the lowest accuracies throughout.

**Table 9:** The mean training and validation time for classifying X-ray images for each deep learning model. All times are in seconds.

Model	NormalScolSpond	NormScol	NormSpond	ScolSpond
SqueezeNet	16.35	14.08	10.99	14.69
GoogLeNet	34.1	26.13	19.24	26.9
Inception-v3	84.58	68.07	47.6	69.5
DenseNet-201	243.52	199.25	126	196.4
MobileNet-v2	151.47	86.1	66.91	99.77
ResNet-101	367.83	284.4	169.87	288.16
ResNet-50	161.04	131.47	78.1	126.98
ResNet-18	62.58	50.28	30.7	49.97
Xception	337.25	221.25	135.68	256.77
Inception-ResNet-v2	246.48	201.2	135.53	210.3
ShuffleNet	97.43	78.8	47.68	81.6
NASNet-Mobile	2271.3	1804.2	1024.5	1764
DarkNet-53	57.5	47.38	31.2	46.69
EfficientNet-b0	215.47	166.25	101.86	170.32

Table 10 shows a comparison to the related work in the literature in terms of performance. Although the related literature produced high accuracy values, these approaches [1, 12, 17] require extensive and error-prone measurement of the biomechanical parameters that indicated the specific disease case, which is not required by our approach. To our knowledge, no other study has included deep learning in the classification of scoliosis vs spondylolisthesis vs normal X-ray images. Colombo et al. [14] addressed the problem of healthy vs scoliosis classification and achieved a low accuracy of 85% at their best. Similarly, Wang et al. [15] could not achieve high accuracy in scoliosis progression detection, and Yang et al. achieved an average accuracy of 80% for distinguishing scoliosis severity based on the Cobb angle (< 10°,10°-19°,20°-44°, or ≥ 45∘). On the other hand, the work in this paper achieves superior accuracy with less input processing/measurements although there is no exactly comparable literature. Nonetheless, the work in this paper can be further improved by:

- Including images of more vertebral column diseases (e.g., disc degeneration, spondylitis, osteoporosis, etc.) in a global image data store similar to ImageNet.
- Development of algorithms and using transfer learning to pinpoint faulty vertebrae or the exact location of the spine anomaly.
- Multistage classification. First images are classified into the corresponding disease state followed by localization or severity grading.
- Continual learning by the development and deployment of mobile applications to aid physicians, collect data, and refinement of the AI models.

**Table 10:** Comparison to the related work in the literature. *healthy, disk herniation, or spondylolisthesis. **Pair-wise permutation of healthy, disk herniation, and spondylolisthesis.

Study	Classification problem	Dataset	Accuracy
Alafeef et al. [1]	Three-class classification*	422 subjects	99.5%
Reshi et al. [12]	Three-class classification*	310 records	99.5%
Unal et al. [17]	Pair-wise**	310 records	96.0%
Colombo et al. [14]	Healthy vs scoliosis	272 scoliosis and 20 healthy	85%
Wang et al. [15]	progressing vs non-progressive scoliosis	490 subjects	76%
Yang et al. [16]	Four classes for scoliosis severity	3640 back images	80%
This work	Three-class and pair-wise classification	331 subjects	96.34%-99.33%

## Conclusion

Artificial intelligence-aided diagnosis systems are being proposed and deployed into many medical areas. These systems have many advantages such as aiding undermanned remote areas, reducing human errors, and optimizing costs. In this paper, it has been shown that transfer deep learning using locally collected X-ray images is able to achieve high performance in terms of correctly identifying normal subjects from those suffering from scoliosis or spondylolisthesis. The highest mean accuracy values ranged from 96.34% for three-class classification to > 97% for the other classification problems. Even though deep learning incurs high overhead, the results show that training and validation can be performed in a reasonably low time using off the shelf hardware resources.

Transfer deep learning can be used to perform spondylolisthesis and scoliosis screening in order to improve the selection of patients who would require further costly CT or MRI imaging. Moreover, the work in this paper can be further improved and made robust by larger databases of more images and more diseases. In addition, field deployment will allow practical benefits and continuous improvements.

## References

[1] Alafeef M, Fraiwan M, Alkhalaf H, Audat Z. Shannon entropy and fuzzy C-means weighting for AI-based diagnosis of vertebral column diseases. Journal of Ambient Intelligence and Humanized Computing. 2019;11(6):2557–2566. doi: 10.1007/s12652-019-01312-3

[2] Konieczny MR, Senyurt H, Krauspe R. Epidemiology of adolescent idiopathic scoliosis. Journal of Children’s Orthopaedics. 2013;7(1):3–9. doi: 10.1007/s11832-012-0457-4 24432052 PMC3566258

[3] Jones J, Thuaimer A. Cobb angle; 2013. Available from: 10.53347/rid-23612.

[4] American Association of Neurological Surgeons. Scoliosis; 2021. Available from: https://www.aans.org/Patients/Neurosurgical-Conditions-and-Treatments/Scoliosis [cited 2022 January 15].

[5] The American Academy of Orthopaedic Surgeons. Spondylolysis and Spondylolisthesis; 2020. Available from: https://orthoinfo.aaos.org/en/diseases--conditions/spondylolysis-and-spondylolisthesis [cited 2022 January 15].

[6] Kalichman L, Kim DH, Li L, Guermazi A, Berkin V, Hunter DJ. Spondylolysis and Spondylolisthesis. Spine. 2009;34(2):199–205. doi: 10.1097/BRS.0b013e31818edcfd 19139672 PMC3793342

[7] Kim H, Kim HS, Moon ES, Yoon CS, Chung TS, Song HT, et al. Scoliosis Imaging: What Radiologists Should Know. RadioGraphics. 2010;30(7):1823–1842. doi: 10.1148/rg.307105061 21057122

[8] Karpiel I, Ziebiński A, Kluszczyński M, Feige D. A Survey of Methods and Technologies Used for Diagnosis of Scoliosis. Sensors. 2021;21(24):8410. doi: 10.3390/s21248410 34960509 PMC8707023

[9] Tu Y, Wang N, Tong F, Chen H. Automatic measurement algorithm of scoliosis Cobb angle based on deep learning. Journal of Physics: Conference Series. 2019;1187(4):042100. doi: 10.1088/1742-6596/1187/4/042100

[10] Horng MH, Kuok CP, Fu MJ, Lin CJ, Sun YN. Cobb Angle Measurement of Spine from X-Ray Images Using Convolutional Neural Network. Computational and Mathematical Methods in Medicine. 2019;2019:1–18. doi: 10.1155/2019/6357171 30996731 PMC6399566

[11] Fu X, Yang G, Zhang K, Xu N, Wu J. An automated estimator for Cobb angle measurement using multi-task networks. Neural Computing and Applications. 2020;33(10):4755–4761. doi: 10.1007/s00521-020-05533-y

[12] Reshi AA, Ashraf I, Rustam F, Shahzad HF, Mehmood A, Choi GS. Diagnosis of vertebral column pathologies using concatenated resampling with machine learning algorithms. PeerJ Computer Science. 2021;7:e547. doi: 10.7717/peerj-cs.547 34395856 PMC8323723

[13] Prasetio RT, Riana D. A comparison of classification methods in vertebral column disorder with the application of genetic algorithm and bagging. In: 2015 4th International Conference on Instrumentation, Communications, Information Technology, and Biomedical Engineering (ICICI-BME). IEEE; 2015. p. 163–168. Available from: 10.1109/icici-bme.2015.7401356.

[14] Colombo T, Mangone M, Agostini F, Bernetti A, Paoloni M, Santilli V, et al. Supervised and unsupervised learning to classify scoliosis and healthy subjects based on non-invasive rasterstereography analysis. PLOS ONE. 2021;16(12):e0261511. doi: 10.1371/journal.pone.0261511 34941924 PMC8699618

[15] Wang H, Zhang T, Cheung KMC, Shea GKH. Application of deep learning upon spinal radiographs to predict progression in adolescent idiopathic scoliosis at first clinic visit. eClinicalMedicine. 2021;42:101220. doi: 10.1016/j.eclinm.2021.101220 34901796 PMC8639418

[16] Yang J, Zhang K, Fan H, Huang Z, Xiang Y, Yang J, et al. Development and validation of deep learning algorithms for scoliosis screening using back images. Communications Biology. 2019;2(1). doi: 10.1038/s42003-019-0635-8 31667364 PMC6814825

[17] Unal Y, Polat K, Kocer HE. Pairwise FCM based feature weighting for improved classification of vertebral column disorders. Computers in Biology and Medicine. 2014;46:61–70. doi: 10.1016/j.compbiomed.2013.12.004 24529206

[18] Mahajan S, Raina A, Gao XZ, Pandit AK. COVID-19 detection using hybrid deep learning model in chest x-rays images. Concurrency and Computation: Practice and Experience. 2021;34(5). doi: 10.1002/cpe.6747

[19] Mahajan S, Raina A, Abouhawwash M, Gao XZ, Pandit AK. Covid-19 Detection from Chest X-Ray Images Using Advanced Deep Learning Techniques. Computers, Materials & Continua. 2022;70(1):1541–1556. doi: 10.32604/cmc.2022.019496

[20] Raina A, Mahajan S, Vanipriya C, Bhardwaj A, Pandit AK. COVID-19 Detection: An Approach Using X-Ray Images and Deep Learning Techniques. In: Lecture Notes in Networks and Systems. Springer Singapore; 2021. p. 7–16. Available from: 10.1007/978-981-16-0695-3_2.

[21] Kokabu T, Kanai S, Kawakami N, Uno K, Kotani T, Suzuki T, et al. An algorithm for using deep learning convolutional neural networks with three dimensional depth sensor imaging in scoliosis detection. The Spine Journal. 2021;21(6):980–987. doi: 10.1016/j.spinee.2021.01.022 33540125

[22] Tan Z, Yang K, Sun Y, Wu B, Tao H, Hu Y, et al. An Automatic Scoliosis Diagnosis and Measurement System Based on Deep Learning. In: 2018 IEEE International Conference on Robotics and Biomimetics (ROBIO); 2018. p. 439–443.

[23] Vergari C, Skalli W, Gajny L. A convolutional neural network to detect scoliosis treatment in radiographs. International Journal of Computer Assisted Radiology and Surgery. 2020;15(6):1069–1074. doi: 10.1007/s11548-020-02173-4 32337647

[24] da Rocha Neto AR, Sousa R, de A Barreto G, Cardoso JS. Diagnostic of Pathology on the Vertebral Column with Embedded Reject Option. In: Vitrià J, Sanches JM, Hernández M, editors. Pattern Recognition and Image Analysis. Berlin, Heidelberg: Springer Berlin Heidelberg; 2011. p. 588–595.

[25] Akben SB. Importance of the shape and orientation of the spine and pelvis for the vertebral column pathologies diagnosis with using machine learning methods. Biomedical Research-India. 2016;27:S337–S342.

[26] Unal Y, Kocer HE. Diagnosis of pathology on the vertebral column with backpropagation and Naive Bayes classifier. In: 2013 The International Conference on Technological Advances in Electrical, Electronics and Computer Engineering (TAEECE); 2013. p. 276–279.

[27] Nguyen TP, Chae DS, Park SJ, Kang KY, Yoon J. Deep learning system for Meyerding classification and segmental motion measurement in diagnosis of lumbar spondylolisthesis. Biomedical Signal Processing and Control. 2021;65:102371. doi: 10.1016/j.bspc.2020.102371

[28] Liao S, Zhan Y, Dong Z, Yan R, Gong L, Zhou XS, et al. Automatic Lumbar Spondylolisthesis_newline Measurement in CT Images. IEEE Transactions on Medical Imaging. 2016;35(7):1658–1669. doi: 10.1109/TMI.2016.2523452 26849859

[29] Cai Y, Leung S, Warrington J, Pandey S, Shmuilovich O, Li S. Direct spondylolisthesis identification and measurement in MR/CT using detectors trained by articulated parameterized spine model. In: Styner MA, Angelini ED, editors. SPIE Proceedings. SPIE; 2017. Available from: 10.1117/12.2254072.

[30] Goodfellow I, Bengio Y, Courville A. Deep Learning. MIT Press; 2016.

[31] Deng J, Dong W, Socher R, Li LJ, Li K, Fei-Fei L. ImageNet: A large-scale hierarchical image database. In: 2009 IEEE Conference on Computer Vision and Pattern Recognition; 2009. p. 248–255.

[32] Zhou B, Lapedriza A, Khosla A, Oliva A, Torralba A. Places: A 10 million Image Database for Scene Recognition. IEEE Transactions on Pattern Analysis and Machine Intelligence. 2017. 10.1109/TPAMI.2017.2723009 28692961

[33] MathWorks^®^. MATLAB^®^-Deep Learning Toolbox; 1994–2022. Available from: https://www.mathworks.com/help/deeplearning/referencelist.html?type=function&s_tid=CRUX_topnav [cited 2022 January 12].

[34] Szegedy C, Liu W, Jia Y, Sermanet P, Reed S, Anguelov D, et al. Going deeper with convolutions. In: 2015 IEEE Conference on Computer Vision and Pattern Recognition (CVPR); 2015. p. 1–9.

[35] Szegedy C, Vanhoucke V, Ioffe S, Shlens J, Wojna Z. Rethinking the Inception Architecture for Computer Vision. In: 2016 IEEE Conference on Computer Vision and Pattern Recognition (CVPR); 2016. p. 2818–2826.

[36] Huang G, Liu Z, Van Der Maaten L, Weinberger KQ. Densely Connected Convolutional Networks. In: 2017 IEEE Conference on Computer Vision and Pattern Recognition (CVPR); 2017. p. 2261–2269.

[37] Sandler M, Howard A, Zhu M, Zhmoginov A, Chen LC. MobileNetV2: Inverted Residuals and Linear Bottlenecks. In: 2018 IEEE/CVF Conference on Computer Vision and Pattern Recognition; 2018. p. 4510–4520.

[38] He K, Zhang X, Ren S, Sun J. Deep Residual Learning for Image Recognition. In: 2016 IEEE Conference on Computer Vision and Pattern Recognition (CVPR); 2016. p. 770–778.

[39] Chollet F. Xception: Deep Learning with Depthwise Separable Convolutions. In: 2017 IEEE Conference on Computer Vision and Pattern Recognition (CVPR); 2017. p. 1800–1807.

[40] Szegedy C, Ioffe S, Vanhoucke V, Alemi AA. Inception-v4, Inception-ResNet and the Impact of Residual Connections on Learning. In: Proceedings of the Thirty-First AAAI Conference on Artificial Intelligence. AAAI’17. AAAI Press; 2017. p. 4278–4284.

[41] Zhang X, Zhou X, Lin M, Sun J. ShuffleNet: An Extremely Efficient Convolutional Neural Network for Mobile Devices. In: 2018 IEEE/CVF Conference on Computer Vision and Pattern Recognition; 2018. p. 6848–6856.

[42] Zoph B, Vasudevan V, Shlens J, Le QV. Learning Transferable Architectures for Scalable Image Recognition. In: 2018 IEEE/CVF Conference on Computer Vision and Pattern Recognition; 2018. p. 8697–8710.

[43] Redmon J. Darknet: Open Source Neural Networks in C; 2013–2016. Available from: http://pjreddie.com/darknet/ [cited 2022 January 12].

[44] Redmon J, Farhadi A. YOLOv3: An Incremental Improvement; 2018. Available from: http://arxiv.org/abs/1804.02767 [cited 2022 January 12].

[45] Tan M, Le Q. EfficientNet: Rethinking Model Scaling for Convolutional Neural Networks. In: Chaudhuri K, Salakhutdinov R, editors. Proceedings of the 36th International Conference on Machine Learning. vol. 97 of Proceedings of Machine Learning Research. PMLR; 2019. p. 6105–6114. Available from: https://proceedings.mlr.press/v97/tan19a.html.

[46] Qian N. On the momentum term in gradient descent learning algorithms. Neural Networks. 1999;12(1):145–151. doi: 10.1016/S0893-6080(98)00116-6 12662723

